# Obscure Tumour—Sudden Death

**Published:** 1854-04

**Authors:** 


*Obscure Tumour—Sudden Death.*

(From a private letter we make the following extract, the case is a singular one, a more complete history would be desirable. Eds. *Journal.*)

An interesting case came partialy under my observation during the past winter, would have been reported to you had any one, or any single set of men had the entire charge of it.

A child three years of age, early last summer had what is called a "stiff neck," with pain in the spinal column, at times application was made to me, and a simple treatment was adopted, by which the pain was relieved and the stiffness obviated. In the middle of the winter I received a summons to see the child and be prepared to remove a tonsil. Before I had made any examination whatever, I learned that they had been to a professed and competent surgeon, who had partially removed the tumor, and that it had grown more rapidly since the removal; they had again applied to him, and he had refused to operate further but gave them medicines, with directions which they had not followed. I then, without examination, refused to do anything for the child, and referred them to the first surgeon. After much entreaty on their part, I examined the case slightly, saw a tumor springing from the fauces, filling the entrance to the throat, and impeding respiration, it was hard to the feel. I did not determine its connections, again I refered them to the first surgeon, promissing to assist him if he thought best to operate and desired. I gave them no opinion whether I thought an operation best. But they still insisted that I should do so, when I told them I would do nothing without further council. This was procured, and the only determination was to return it to the first surgeon; which we finally persuaded them to do. The first surgeon would not operate, but they found two physicians who finally consented to try, all having expressed the opinion that the operation would be dangerous if not fatal. A cork was placed between the teeth, and as yet no instrument had touched the child, it fainted and died. I regret that the tumor with its connections was not removed. I have been thus particular in the historical paart of the transaction, to show that a correct report could not easily be made. I was not present at the operation.

Query. what was the tumor? In this doctors disagree. Let us examine the circumstances. The tumor filled the fauces and partially the mouth, so completely that owing to the difficulty of breathing, it was scarcely possible to determine its connections, whether fauces tonsil or tongue. There appeared no enlargement of the opposite side, or redness of the surrounding parts. The submaxillary glands on the affected side were enlarged but not on the other. After the operation there was no hemorrhage, the operating surgeon after the death of the child, found the connections clearly joined to the tongqe with a wide short base or neck. The tumor was very brittle, holding a tenaculum but tearing out with the least force, internally it was of a fleshy character, rather colored, but hardly sarcomatous. Some regarded it as malignant and others as a polypus.* Those who examined it the most carefully, regard it as the latter. The tumor had not been fully removed at the time of the operation, and grew very rapidly afterwards.—Would it have been advisable to have performed tracheotomy before commencing the operation.

Truly Yours,

STORRS HALL.

[We learn from our correspondent that a medical society has been formed by the physicians of the county of Fond du Lac.—Dr. Wm H. Walker is the delegate to the American Medical Association. EDS. JOURNAL.]

---

The friends of Prof. Brainard will be gratified to know that he is reaping new honors in the old world. We present our readers in this number, an interesting letter from Prof. B., also a translation from the Gazette des Hopiteaux of his experiments on vegetable poisons. He is expected home about the 20th inst., J.

*In Chelius surgery is given a description of polyps of almost a similar character, but situated lower in the throat than this.

